# Does Chinese culture influence psychosocial factors for heroin use among young adolescents in China? A cross-sectional study

**DOI:** 10.1186/1471-2458-10-563

**Published:** 2010-09-21

**Authors:** Hongjie Liu, Jian Li, Zhouping Lu, Wei Liu, Zhiyong Zhang

**Affiliations:** 1 Department of Epidemiology and Community Health, School of Medicine, Virginia Commonwealth University, Richmond, VA, USA; 2 School of Public Health, Guangxi Medical University, Nanning, Guangxi, China; 3 Guangxi Center for Disease Control and Prevention, Nanning, Guangxi, China

## Abstract

**Background:**

Little empirical research has examined how cultural factors influence psychosocial factors for heroin drug use. The objectives of the study were to investigate the levels of individualism and collectivism among young adolescents and how cultural differences were associated with the constructs of the Theory of Planned Behavior and other psychosocial factors for heroin drug use.

**Methods:**

A cross-sectional study was conducted among young adolescents in an HIV and heroin-stricken area in China. The Individualism-Collectivism Interpersonal Assessment Inventory (ICIAI) was used to measure cultural norms and values in the context of three social groups: family members, close friends, and classmates.

**Results:**

A total of 220 boys and 241 girls were recruited and participated in an interview. Compared to boys, girls reported higher levels of the three specific-relationship ICIAIs, as well as higher levels of perceived behavioral control for heroin use, perceived peer control, and communication with parent about heroin use, but a lower level of favorable attitude towards heroin use. The levels of descriptive and subjective norms of heroin use were low in both girls and boys. Among boys, family ICIAI was positively associated with perceived behavioral control, and friend ICIAI was positively associated with perceived peer control and communication with parent. Among girls, family ICIAI was positively associated with perceived behavioral control and communication with parents about heroin use, but negatively with favorable attitudes to heroin use; friend ICIAI was positively associated with perceived peer control, and classmate ICIAI was negatively associated with favorable attitudes toward heroin use.

**Conclusions:**

This study documents that collectivistic aspects of Chinese culture may influence psychosocial factors for heroin use, although the patterns are varied by gender. Findings provide an empirical basis for the development of culturally competent intervention programs for heroin use intervention and prevention.

## Background

Culture has been considered an ecological-level variable that may influence mental processes, human behaviors, and health [1]. Although the goals of prevention of diseases and maintenance of health may be the same across cultures, cultural differences exist in the perception of problems, in psychological reactions to the problems, and in preferred strategies for coping with them [2]. Logically, theoretical models or approaches to prevent risk behaviors may prove insensitive or inappropriate when applied across countries with different cultures. As an ecological-level variable, culture has been shown to influence human behaviors through shaping and molding individuals' psychological processes [2].

The majority of the theoretical models of behavioral change have been developed and designed for assessing HIV-related risks among people in Western countries. One of the commonly-used models in HIV behavioral intervention is the theory of planned behavior (TPB) [3]. The theory posits that the intention to perform or to not perform a behavior is the immediate determinant of the behavior. Intention, in turn, is a direct function of three determinants - an individual's attitudes towards the behavior, norms regarding the behavior (i.e., social pressure to perform or not to perform the behavior), and perceived behavioral control (i.e., the perceived capability of performing the behavior). In addition to the constructs of the theory of planned behavior, research has also demonstrated that better communication with parents about a behavior and perceived peer control for preventive practices are two important psychosocial factors for individuals' maintenance of health [4-6]. Success of the theory of planned behavior in the examination of drug use has been documented in many studies conducted in Western countries where individualistic cultures dominate.

Individualism versus Collectivism (IC) is the most common dimension of culture operationalized at the individual level [7,8]. Individuals in collectivistic cultures tend to see themselves as interdependent with their groups and usually behave according to social norms or regulations. People are embedded and situated within certain roles and statuses in collectivistic society and are advised to exercise emotional restraint to avoid shame and maintain public dignity [9]. In contrast, individuals in individualistic cultures see themselves as independent of groups and generally behave according to personal needs and choices. People in individualistic cultures are motivated by their own interests, which are often valued over the interests of their groups. Both individualistic and collectivistic values within Chinese culture are endorsed although Chinese culture is more collectivistic than individualistic [10].

Matsumoto and colleagues developed the Individualism-Collectivism Interpersonal Assessment Inventory (ICIAI) to measure IC at the individual level [11]. ICIAI enables evaluation of the individualism vs. collectivism levels as well as subjects' perception of IC values related to interacting with people in four types of relationships: family, close friends, colleagues and strangers. According to Matsumoto, "values are concepts or beliefs about desirable end states or behaviors that guide our selection of behaviors and evaluation of events" [12]. An individual could have collectivistic tendencies (or perceived values) with family members or close friends, but individualistic tendencies with colleagues or strangers.

There are few studies examining whether the individualistic-oriented intervention theories are sensitive to the collectivistic-oriented culture and how cultural differences influence psychosocial factors for heroin use. We, thus, conducted a cross-sectional study among middle-school students in an HIV- and heroin-stricken area in China. The objectives of the study were to investigate the levels of individualism and collectivism among young adolescents and how cultural differences were associated with the constructs of the theory of planned behavior, communication with parents about heroin use, and perceived peer control for preventive practices. We expected that girls, compared to boys, had higher levels of collectivistic tendencies. This expectation was based on gender-preferences in China. Traditionally, males are socialized to be independent, self-reliant, and emotionally detached, whereas females are expected to be dependent, reliant and emotional [2]. Illicit drug use is considered to be non-conforming to family or group values as it is not socially accepted in Chinese culture. In order to save family face and the group dignity, people may try to stay away from heroin use. We thus hypothesized that individuals with higher levels of collectivistic tendencies possessed lower levels of favorable attitudes toward heroin use and higher levels of perceived behavioral control of heroin use, subjective and descriptive norms regarding heroin use, communication with parents, and perceived peer control.

## Methods

### Study site

We selected "H County" in Guangxi province as the study site. Guangxi is along a drug trafficking route, which originates from the "Golden Triangle", passes through northern provinces of Vietnam and Guangxi and finally into Hong Kong and the rest of the world [13]. Guangxi ranks second among China's 31 provinces in terms of the estimated number of people living with HIV/AIDS in 2007 [14]. Similar to the national epidemic of HIV, the majority of infected cases are from rural areas. H County is one of the most HIV and heroin-stricken rural areas in Guangxi. Although the actual number of heroin users is unknown, it is estimated that there are between 8,000 to 10,000 in the county, 70% of whom share injection needles. According to the local surveillance report, the estimated HIV prevalence among heroin users was between 25-30% [15].

### School selection and participant eligibility

Since the goal of the study was to investigate how individualism and collectivism influenced psychosocial factors for heroin use, we purposely selected middle schools located in the areas where prevalence of heroin injection use was high. There were 66 middle schools in H County. The following steps were taken to select schools: (1) ranked all rural towns based on the levels of heroin injection prevalence, using local surveillance data; (2) selected the top five towns in the ranking; (3) randomly selected one school from each town if there was more than one school; (4) randomly selected two schools out of five schools for qualitative studies and the remaining three schools for a cross-sectional study. Eligibility of participants included: (1) students in second and third grade (equivalent to the seventh and eighth grade in the US) in the three selected middle schools, and (2) students who were able to obtain parental permission and to invite one of their parents or guardians to participate in the study.

We first selected classes that had enough students in each of the three schools. If the number of students was not enough because students could not obtain their parental permission or could not invite one of their parents or guardians to participate in the study, students in other classes in the same grade were invited. All students who obtained parental permission and invited one of their parents or guardians participated in the study. The study protocol was approved by the Institutional Review Boards of Virginia Commonwealth University and Guangxi Medical University.

### Interview

Students used a self-administered questionnaire to answer questions in classrooms in each selected middle school. Only one student was permitted to sit at a desk to answer questions on the anonymous questionnaire (two students share one desk in Chinese schools in rural areas). One or two trained interviewers presented in the classrooms and answered questions raised by students. All interviewers received training in interviewing techniques, developing rapport, ensuring confidentiality, and answering questions raised by subjects.

### Questionnaire and Measures

Measurement items and scales were initially drafted in English and then translated into Chinese by three research members who were fluent in both languages. The Chinese version of measures was then distributed to research team members who reviewed and modified wording to make them appropriate for the Chinese context. Next, the self-administered questionnaire was piloted among students in a second-grade classroom in a middle school (all students in the classroom were invited to participate). After completion of the questionnaire, we asked students to make comments or suggestions, focusing on understanding and wording of the questions listed in the questionnaire. Some modifications were made based on the results of the pilot.

#### Individualism-collectivism (IC)

Matsumoto's Individualism-Collectivism Interpersonal Assessment Inventory (ICIAI) was used to measure IC at the individual level [11]. This instrument was considered to have two major strengths that could contribute to this study. First, it focuses on interpersonal interactions in different social relationships and allows assessment of the relative importance of individualism or collectivism within these relationship types. Second, studies conducted in a wide range of ethnic groups have demonstrated high levels of validity and reliability [16,17]. The original ICIAI is a 19-item scale measuring individual IC differences in rating values and behaviors related to social interaction in four social groups. Two of the social relationships are in-groups (family members and close friends); the other two are out-groups (colleagues and strangers). Before using the instrument, we qualitatively evaluated its acceptability in in-depth interviews with 18 students and 18 of their parents. Modifications were made based on input received from these interviews. First, the scope of the inventory was limited to the values rated by subjects in three types of relationships: family members, close friends, and classmates. The relationship of "colleagues" was replaced by the group of "classmates" and the group of "strangers" was removed as it was very difficult to rate values on those with whom students did not have any contacts. Second, one item was deleted from the instrument because the meaning of the item could not be appropriately expressed and translated into Chinese (the item was "accept award, benefits, or recognition based only on age or position rather than merit from them."). Thus, a total of 54 items (18 items × 3 relationship groups) were used to measure the levels of IC tendencies towards three interaction relationships, e.g., family members, close friends, and classmates (sample items: 'Share credit for their accomplishments'; 'Share blame for their failures'; 'Be loyal to them'; 'Sacrifice your goals for them'; 'Maintain harmonious relationships with them'; 'Follow norms established by them'). Family members were defined as the core, nuclear family that was present during a student's growing years, such as his/her parents and brothers or sisters. Close friends were defined as those individuals whom students considered "close;" i.e., with whom they spend a lot of time after class and/or seek for help or advice. Classmates were students with whom study subjects interact on a regular basis, but with whom they were not be particularly close. This category did not include classmates that were considered as 'close friends'.

Students were requested to rate how important each of the item as a value to them in a five5-point scale ranging from 1 'not important at all' to 5 'very important'. The score of each of the three relationship-specific ICIAI value scales (family ICIAI, friend ICIAI, and classmate ICIAI) were created by averaging the items within each relationship group. Higher scores reflected more collectivism.

#### Psychosocial factors for heroin use

Measurement scales of the following psychosocial constructs were adopted from reports by Rice et. al. [18], Orbel S et. al. [19], Jaccard, J et. al. [20], and Jessor, R et. al. [21].

*Favorable attitude towards heroin use *was measured by a seven-item scale (e.g., using heroin would make me feel cool). Response options for these items ranged from 0 (strongly disagree) to 3 (strongly agree). *Perceived behavioral control *was measured by a ten-item scale (e.g., I am confident that I could resist peer pressure to use heroin). Response choice to each item was from 0 (strongly disagree) to 3 (strongly agree). *Descriptive norms *refer to students' perceptions of other people's behaviors (heroin use) [22] and were acquired by asking students whether they knew family members, classmates, or villagers who were heroin users (0: no; 1 yes). *Subjective norms *(the perceived social pressure to use or not to use heroin) were assessed by asking whether students perceived that their family members/classmates would encourage them to try heroin (0: no; 1 yes). *Perceived peer control *was measured by six items (e.g., If you were doing something that is bad for your health, e.g., use heroin, would your friends try to get you to stop?). Response choice to each item ranged from 0 (definitely would not) to 3 (definitely would). *Communication with parent about heroin use *was assessed by a ten-item scale (e.g., I can talk with my parents when I have concerns or questions about heroin use). Possible answers ranged from 0 (strongly disagree) to 3 (strongly agree).

### Analysis

In descriptive analyses, t-test and chi-square test were used to compare the differences in ICIAIs and psychosocial factors for heroin use between boys and girls. A repeated measures analysis of variance (ANOVA) was used to examine the within-subject differences in the three relationship-specific ICIAIs. Multiple linear regression and logistic regression analysis were performed to examine the relationships of the three specific-relationship ICIAIs and psychological variables, adjusting for age, grade and ethnicity. Because very few students reported descriptive and subjective norms of heroin use, except for descriptive norms of heroin use by villagers, the relation was only examined between ICIAIs and descriptive norms of heroin use by villagers in a logistic regression model. Statistical analyses were done using SAS (version 9.1).

## Results

### Internal consistency of measurement

Internal consistency of the measurement scales was assessed using Cronbach's coefficient alpha. Among boys, the alpha of ICIAIs and psychosocial measurement scales ranged from 0.77 to 0.90. Among girls, it ranged from 0.71 to 0.88 (Table 1).

**Table 1:** Reliability of ICIAIs and psychosocial measurement scales

Scales	Items	Cronbach's alpha	
		
		Boys	Girls
Family ICIAI	18	0.90	0.88
Friend ICIAI	18	0.87	0.87
Classmate ICIAI	18	0.85	0.88
Favorable attitude towards heroin use	7	0.84	0.78
Perceived behavioral control	10	0.86	0.81
Perceived peer control	6	0.77	0.71
Communication with parent about heroin use	10	0.80	0.83

### Description of the study sample and measurements

A total of 220 boys and 241 girls were recruited and participated in an interview. The average age of the participants was 14.9 years [standard deviation (SD) = 0.78, range 13-18 years old]. Two thirds of the students were in the second grade and 37% in the third grade in the selected middle schools. Most of subjects were members of the Han majority (Table 2).

**Table 2:** Comparisons of demographic, ICIAIs and psychosocial variables between boys and girls

	Boys (n = 220) **Mean (SD**^**a**^**) or N (%)**	Girls (n = 241) Mean (SD) or N (%)	**P - value**^**b**^
Age	14.90 (0.84)	14.87 (0.72)	0.77
Grade			
Seventh	88 (40)	79 (33)	0.10
Eighth	130 (60)	160 (67)	
Ethnicity			
Han	174 (79)	191 (79)	0.97
Zhuang and others	46 (21)	50 (21)	
			
Family ICIAI	3.56 (0.71)	3.77 (0.64)	**<0.01**
Friend ICIAI	3.19 (0.62)	3.39 (0.60)	**<0.01**
Classmate ICIAI	2.64 (0.60)	2.84 (0.67)	**<0.01**
Favorable attitude towards heroin use	3.49 (3.16)	2.33 (2.51)	**<0.01**
Perceived behavioral control	26.31 (4.48)	27.08 (3.15)	**0.04**
Descriptive norms of heroin used by family members			
No	219 (100)	233 (97)	**0.01**
Yes	0 (0)	8 (3)	
Descriptive norms of heroin used by villagers			
No	98 (45)	84 (35)	**0.03**
Yes	121 (55)	156 (65)	
Descriptive norms of heroin used by classmates			
No	216 (98)	237 (98)	1.00
Yes	4 (2)	4 (2)	
Subjective norms (perceived encouragement of heroin use by family members)			
No	217 (100)	241 (100)	
Yes	0 (0)	0 (0)	
Subjective norms (perceived encouragement of heroin use by classmates)			
No	216 (99)	239 (100)	0.61
Yes	2 (1)	1 (0)	
Perceived peer control	12.93 (3.03)	14.93 (2.26)	**<0.01**
Communication with parents about heroin use			
	29.90 (4.28)	31.39 (4.34)	**<0.01**

^a ^Standard deviation

^b ^p - value ≤ 0.05 in bold

Compared to boys, girls reported higher levels of the three specific-relationship ICIAIs, as well as higher levels of perceived behavioral control, perceived peer control, and communication with parent about heroin use, but a lower level of favorable attitude towards heroin use (Table 2). The levels of descriptive norms and subjective norms were very low in both girls and boys, except for the descriptive norms of heroin use by villagers.

The repeated measures analysis of variance (ANOVA) documented that the within-subject differences in the three relationship-specific ICIAIs were statistically significant among boys (F = 203.68, p < 0.01) and girls (F = 284.32, p < 0.01). Both boys and girls exhibited the highest values in the family ICIAI, high values in the friend ICIAI, and lower values in the classmate ICIAI (Table 2).

### Associations between relationship-specific ICIAIs and psychosocial variables

Table 3 presents results from multiple linear regression and logistic regression analyses. Among boys, the family ICIAI scale was positively associated with perceived behavioral control. Friend ICIAI was positively associated with perceived peer control and communication with parent about heroin use.

**Table 3:** Associations between ICIAIs and psychosocial variables in boys and girls

	Boys		Girls	
	
	**β**^**a**^	**95%CI**^**b**^	β	95%CI
Favorable attitude towards heroin use				
Family ICIAI	-0.36	-1.10 - 0.38	**-0.90**	**-1.60 **- **-0.20**^c^
Friend ICIAI	-0.10	-1.09 - 0.89	0.25	-0.70 - 1.20
Classmate ICIAI	-0.55	-1.41 - 0.31	**-0.65**	**-1.30 **- **-0.01**
Age	0.12	-0.50 - 0.75	-0.28	-0.80 - 0.24
Grade	0.29	-0.78 - 1.35	0.35	-0.43 - 1.14
Ethnicity	0.55	-0.52 - 1.62	0.10	-0.68 - 0.88
Perceived behavioral control of heroin use				
Family ICIAI	**1.34**	**0.32 - 2.36**	**1.33**	**0.43 **- **2.23**
Friend ICIAI	0.29	-1.08 - 1.65	-1.02	-2.24 - 0.20
Classmate ICIAI	-0.87	-2.06 - 0.32	0.37	-0.46 - 1.20
Age	**-1.13**	**-1.99 **- **-0.28**	0.05	-0.62 - 0.72
Grade	**2.04**	**0.57 **- **3.50**	0.31	-0.69 - 1.32
Ethnicity	1.05	-0.43 - 2.52	0.26	-0.74 - 1.26
Perceived peer control				
Family ICIAI	0.26	-0.44 - 0.97	-0.01	-0.64 - 0.62
Friend ICIAI	**1.05**	**0.11 **- **2.00**	**0.95**	**0.10 **- **1.80**
Classmate ICIAI	-0.70	-1.52 - 0.12	-0.32	-0.90 - 0.25
Age	-0.27	-0.87 - 0.32	-0.10	-0.56 - 0.37
Grade	0.35	-0.66 - 1.37	-0.33	-1.03 - 0.37
Ethnicity	0.21	-0.81 - 1.23	**0.95**	**0.26 **- **1.65**
Communication with parent about heroin use				
Family ICIAI	0.37	-0.62 - 1.35	**1.68**	**0.50 **- **2.86**
Friend ICIAI	**1.47**	**0.15 **- **2.78**	-0.36	-1.96 - 1.24
Classmate ICIAI	-0.48	-1.64 - 0.67	**1.16**	**0.08 **- **2.25**
Age	-0.44	-1.27 - 0.40	0.52	-0.36 - 1.39
Grade	0.37	-1.05 - 1.79	-1.19	-2.52 - 0.13
Ethnicity	0.74	-0.69 - 2.17	-0.25	-1.56 - 1.07

^a ^Regression coefficient in multiple linear regression

^b ^95% confidence interval of regression coefficients or odds ratios

^c ^p - value ≤ 0.05 in bold

^d ^Odds ratios in multiple logistic regression

Among girls, family ICIAI was positively associated with perceived behavioral control and communication with parents regarding heroin use, but negatively associated with favorable attitude towards heroin use. Friend ICIAI was positively associated with perceived peer control. Classmate ICIAI was positively associated with communication with parents about heroin use, but negatively associated with favorable attitude towards heroin use. Descriptive norms of heroin use were not significantly associated with any ICIAI items.

## Discussion

This study documents that Chinese collectivistic culture may influence risky psychosocial factors for heroin use among young adolescents in China. Collectivistic aspects of Chinese culture appeared to inhibit these risk factors for heroin use, although the patterns varied by gender. The findings provide an empirical basis for developing culturally competent intervention programs for heroin use. To our knowledge, this is the first study quantitatively examining how cultural factors influence psychosocial factors for heroin use in China.

The results suggest that Chinese young adolescents show some variations in the degree to which they have internalized traditional Chinese collectivistic culture. The levels of the three relationship-specific ICIAIs in girls were significantly higher than were in boys, and indicate that girls were more collectivistic than boys. The gender differences may reflect traditional gender-role differentiations. The gender differences in ICIAIs may indicate parental expectations and influences in China. Chinese parents expect boys to be autonomous and be the major financial and social supporters in households (the head of the household), while girls are expected to be conforming and obedient to parents before marriage, to their husband after marriage, and to their son after the death of their husband. These parental expectations prevail to a greater degree in rural areas as more traditional and conservative cultural patterns are more common there compared to urban areas.

As reflected by the different degree of the three relationship-specific ICIAIs, both boys and girls exhibited the highest values toward family members, and reported more collectivistic attitudes regarding close friends compared with classmates. This finding supports Matsumoto and colleagues' argument that if a culture fosters collectivistic tendencies within relationships between the self and in-groups (e.g., family members or close friends), it is unlikely that those same tendencies would occur to the same degree in relationships with members of out-groups (e.g., classmates) [11]. Chinese collectivistic culture has long been influenced by the traditions of Confucius, Taoism and Buddhism, among which Confucianism has been most influential in shaping values and behavior patterns and structures of the family and community [23]. Confucianism posits the family as the fundamental unit of society with interdependent responsibilities and expectations. In the Confucian tradition, family is the irreducible unit rather than the individual, and members are expected to be involved closely with other family members' lives [24]. The higher value tendencies toward family also indicate the importance of the family's role in child development. Studies have demonstrated that this parent-dominated relationship lessens somewhat over time with the development of peer relationship. For example, Helsen and colleagues reported that adolescents oriented themselves increasingly to peers and friends between the ages of 16-18 years of age at which time they were regarded as having an importance equal to that of the parents [25]. The mean age of this study sample was 15 years, and it was not surprising that they showed higher levels of collectivistic tendencies towards family members and close friends in comparison to classmates. It is possible that when they move to high schools (at the age of 16 years old), they may consider peer relationship equally, or perhaps even more important than parental relationship.

Findings of this study document that the relationship-specific ICIAIs were associated with several constructs of the theory of planned behavior, with variations by gender. Boys and girls who retained stronger collectivistic tendencies towards family members were more likely to perceive abilities to perform positive activities against heroin use. The perception may result from characteristics of collectivistic culture, i.e. collectivistic individuals preserve family values and believe that it is their responsibility, as a family member, to save the family's face and maintain the family's dignity. Avoidance of damage to their family values may motivate and enhance their ability to avoid heroin use. Perceived behavioral control is considered a key construct in the theory of planned behavior and acts as a moderator of the relationships of attitudes and norms with intention [26]. Interestingly, favorable attitudes towards heroin use were not significantly associated with any of the three specific-relationship ICIAIs among boys. Among girls, favorable attitudes towards heroin use were significantly negatively associated with family ICIAI and classmate ICIAI. According to the theory of planned behavior, attitudes towards a behavior reflect the degree to which performance of a behavior is positively or negatively valued. Since girls were more collectivistic than boys, their family's values may lead to more negative evaluation of heroin-use consequences than might be the case if classmates opinions had been valued more. The findings that family ICIAI and classmate ICIAI were inversely associated with attitudes towards heroin use among girls and no such significant associations occurred among boys may help explain why girls' attitudes towards heroin use were less favorable than boys. We found that descriptive norms of heroin use by villagers were not associated ICIAI in either boys or girls. Adolescents may think that heroin use by villagers was the business of those villagers and not an influence on their own behavior. Because few students reported subjective norms, it prevented us from examining the relationships with ICIAIs.

As expected, perceived peer control for performance of acceptable practices and behaviors was positively associated with friend ICIAI among girls and boys. Triandis [8] has suggested that collectivistic people tend to have few relationships that are intimate but profound in that intimacy, whereas individualistic people have many relationships of low intimacy. In order to maintain intimacy and harmony, collectivists are more likely to follow peers' norms, controls, or instructions than their individualistic counterparts. Because girls in this study exhibited greater values towards friends than boys did, they perceived higher levels of peer control than boys did. We expected that adolescents with greater values towards family would be more likely to talk and exchange ideas about heroin use with parents; however, this only occurred among girls. Our study also found that communication with parents about heroin use was significantly associated with friend ICIAI in boys and classmate ICIAI in girls. Explanations and mechanisms to account for these associations are not clear. Further research, especially qualitative research, is needed to examine these unexpected findings.

There are limitations that should be noted in the study. Because the study participants were recruited from HIV and heroin-stricken rural areas in one county, the sample was not representative of all rural areas in China. Future large scale studies need to be done to verify findings from this study. Another limitation is that we could not examine relationships between the three determinants of the intention to use heroin because very few students (1%) reported such intention although heroin use is a serious problem in that area. The low level of intention may be due to the fact, according to available research, that most of Chinese heroin users started using it between the age of 16-24 years old often when they dropped out of middle schools [27].

## Conclusions

Taking the findings together, this study suggests how Chinese culture can be integrated with concepts from the theory of planned behavior and other risk and protective factors for drug use that have been developed in western countries. The findings indicate that interventions aimed at preventing heroin-use initiation among adolescents should target both adolescents and their parents. As values towards close friends also play an important role in the theory of planned behavior, interventions that include close peers such as the popular opinion leader intervention approach may be useful [28,29]. The contents of interventions should include components to promote and enhance collectivistic values towards family and friends.

## List of abbreviations used

IC: Individualism versus Collectivism; ICIAI: Individualism-Collectivism Interpersonal Assessment Inventory; IDU: injection drug use; PBC: perceived behavioral control; SD: standard deviation; TPB: the theory of planned behavior.

## Competing interests

Funding for this study was awarded to Hongjie Liu by NIH-NIDA Grant R03DA019397. The NIDA had no further role in study design; in the collection, analysis and interpretation of data; in the writing of the report; or in the decision to submit the paper for publication. All authors declare that they have no conflicts of interest.

## Authors' contributions

HL, ZL and WL designed the study and wrote the protocol. JL and ZZ managed the literature searches and summaries of previous related work. Authors HL and JL undertook the statistical analysis and wrote the manuscript. All authors contributed to and have approved the final manuscript.

## Pre-publication history

The pre-publication history for this paper can be accessed here:

http://www.biomedcentral.com/1471-2458/10/563/prepub
